# Modelling altered signalling of G-protein coupled receptors in inflamed environment to advance drug design

**DOI:** 10.1038/s41598-023-27699-w

**Published:** 2023-01-12

**Authors:** Arne Thies, Vikram Sunkara, Sourav Ray, Hanna Wulkow, M. Özgür Celik, Fatih Yergöz, Christof Schütte, Christoph Stein, Marcus Weber, Stefanie Winkelmann

**Affiliations:** 1 Zuse Institute Berlin, 14195 Berlin, Germany; 2 Institut für Mathematik und Informatik, Freie Universität Berlin, 14195 Berlin, Germany; 3 Institute of Experimental Anaesthesiology, Charité Universitätsmedizin, Corporate Member of Freie Universität and Humboldt Universität Berlin, 12200 Berlin, Germany

**Keywords:** Cell biology, Computational biology and bioinformatics, Drug discovery, Systems biology, Mathematics and computing

## Abstract

We previously reported the successful design, synthesis and testing of the prototype opioid painkiller NFEPP that does not elicit adverse side effects. The design process of NFEPP was based on mathematical modelling of extracellular interactions between G-protein coupled receptors (GPCRs) and ligands, recognizing that GPCRs function differently under pathological versus healthy conditions. We now present an additional and novel stochastic model of GPCR function that includes intracellular dissociation of G-protein subunits and modulation of plasma membrane calcium channels and their dependence on parameters of inflamed and healthy tissue (pH, radicals). The model is validated against in vitro experimental data for the ligands NFEPP and fentanyl at different pH values and radical concentrations. We observe markedly reduced binding affinity and calcium channel inhibition for NFEPP at normal pH compared to lower pH, in contrast to the effect of fentanyl. For increasing radical concentrations, we find enhanced constitutive G-protein activation but reduced ligand binding affinity. Assessing the different effects, the results suggest that, compared to radicals, low pH is a more important determinant of overall GPCR function in an inflamed environment. Future drug design efforts should take this into account.

## Introduction

The family of G-protein coupled receptors (GPCRs) represents the largest class of receptors in the human genome and some of the most common drug targets. Located on the cell membrane, they transduce extracellular signals into key physiological effects. Natural GPCR ligands include neurotransmitters, chemokines, hormones, odours or photons. GPCRs are involved in a large number of disorders, such as diabetes, high blood pressure, depression, addiction, pain, arthritis, Parkinson’s and many others^1^. A prominent member of this family is the $\mu$-opioid receptor (MOR). It binds endogenous opioid peptides (e.g. endorphins, enkephalins) as well as exogenous drugs (e.g. morphine, fentanyl), and its activation results in the modulation of intracellular G-proteins and second messengers (e.g. cAMP, ion channels). MOR plays major roles in analgesia (inhibition of pain), addiction, bowel movement, arousal, respiration and other physiological functions^2^.

Recent works of our group^3^ led to the development of the novel analgesic compound *N*-(3-fluoro-1-phenethylpiperidin-4-yl)-*N*-phenylpropionamide (NFEPP) which activates the MOR preferentially at acidic extracellular pH-levels, as given in injured tissues^2^. This is of utmost interest because it may preclude the adverse effects of conventional MOR agonists like fentanyl which include constipation, sedation and apnea. These adverse effects are mediated mostly in the brain and the gut, i.e. in healthy tissues (pH 7.4). Since the generation of pain can be effectively inhibited by blocking the electrical excitation of sensory neurons at the site of the injury (i.e. the origin of nociceptive stimulation), this gives rise to the hope that NFEPP might have less or even no adverse effects, which could already be corroborated in animal studies^3–6^.

Up to now, the effects of NFEPP and fentanyl were mathematically analysed at the level of their binding rates at relevant amino acid residues accessible from the extracellular side of MOR^3,7^. To get a more complete picture, we herein present a model of the intracellular second messenger pathways relevant to pain and analgesia. The mechanism underlying the analgesic effect of MOR activation in nociceptive neurons is mainly due to a stabilisation or even lowering of the plasma membrane potential beneath the threshold value required to elicit an action potential^2,8^. This effect is mediated via intracellular inhibitory G-proteins, which dissociate into $\alpha$- and $\beta \gamma$-subunits after formation of a receptor–ligand complex^9^. Among other actions, the $\beta \gamma$-subunits bind to calcium channels in the plasma membrane. This leads to closure of the channels, thereby lowering the amount of positive calcium-ion influx and reducing cellular excitability^2,8,10,11^.

**Figure 1:**
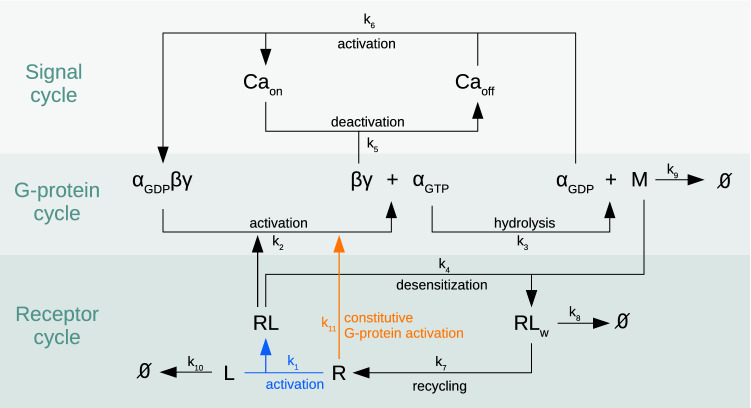
Overview of the reaction network. Biochemical reaction network for the $\mu$-opioid receptor signalling pathway, connecting the receptor cycle to the G-protein cycle and further to the signal cycle of membrane calcium channel modulation, see “The reaction network” for an explanation. The focal point of this study is the analysis of the impact that the rates for ligand-induced receptor activation (blue) and for constitutive G-protein activation (orange) have onto the overall dynamics. The values of the rate constants $k_j$ can be found in Table  2.

In this paper, we model this pathway to analyse the in vitro effects of fentanyl and NFEPP on the number of closed membrane calcium channels and activated (i.e. dissociated) G-protein complexes at different pH-levels in cultured cells and sensory neurons to investigate basic mechanisms underlying opioid analgesia. We propose a reaction network that connects the receptor–ligand interactions to the G-protein cycle, and further to the signalling cycle of calcium channel opening and closing (see Fig. 1 for an illustration). After careful parametrization and validation of the model using Förster resonance energy transfer (FRET) data from corresponding in vitro experiments, the stochastic reaction process was simulated for different values of the receptor–ligand binding rate, comparing the mean inhibition of calcium currents resulting from these numerical simulations to additional data from in vitro patch clamp experiments. By numerical simulation of the reaction network, we observe that the binding rate has a non-linear effect onto the mean amplitude of deactivated calcium channels, which explains the different effects of NFEPP and fentanyl in inflamed versus healthy environments.

It is important to note that our approach differs from others that have investigated signalling pathways from receptor to the nucleus or to intracellular second messengers (not to the plasma membrane)^12,13^. In contrast to those studies, we choose a stochastic approach because it delivers more information than deterministic alternatives.

Aside from pH, other inflammatory mediators play important roles. For example, reactive oxygen species (radicals) can modulate the function of GPCRs^14–17^. In order to understand the interplay between pH and additional radicals for the signalling, we modelled different scenarios and performed in vitro experiments. In the precense of radicals, G-protein activation can be initiated in the absence of an opioid ligand (so-called constitutive G-protein activation). Motivated by this observation, we included the reaction of constitutive G-protein dissociation in our network.

Altogether, the resulting model allows to study two different inflammatory conditions:

- influence of pH, and
- influence of radicals.

With regard to (a), lower pH value changes the protonation state of amino acid residues and opioid ligands, and we investigate whether this affects the binding rates and subsequent modulation of calcium channels. Concerning (b), we study whether an increased concentration of radicals may have an effect on the binding affinity of ligands and/or increase the probability for constitutive G-protein dissociation.

A crucial advantage of the model is that it also permits to analyse the combined effects of (1) and (2), and our results suggest that, compared to radicals, low pH is a more important determinant of overall GPCR function in an inflamed environment.

Unlike in our previous work, we here studied these effects at the systems biology instead of molecular level: results from reaction network simulation are integrated with data from in vitro experiments in order to analyse the consequences of environment-dependent ligand binding rates onto the downstream signalling, i.e. calcium channel inhibition.

## Models and methods

In this section we introduce a probabilistic model for the signalling pathway from receptor activation over the G-protein cycle to the calcium channel inhibition. We explain the in vitro experiments which were performed to validate the modelling results and estimate the parameter values. Moreover, we motivate our choice of parameter values and specify the numerical approach used for solving the system.

### The reaction network

The biochemical reaction network under consideration consists of the following reactions (see Table 1 for an overview and Fig. 1 for an illustration). A ligand *L* attaches to a receptor *R* in the membrane, resulting in a receptor–ligand complex *RL* (reaction ${\mathcal {R}}_1$). This binding process is dependent on the concentration of protons (i.e. pH) in the microenvironment of the receptor. The protonation of both the ligand and certain residues in the receptor are important determinants for receptor activation, likely due to the pH-dependent formation of hydrogen bonds and/or salt bridges between ligand and receptor^18,19^.

This receptor–ligand complex *RL* activates a trimeric G-protein complex which leads to exchange of *GDP* by *GTP* and subsequent dissociation into $\alpha$- and $\beta \gamma$-subunits (reaction ${\mathcal {R}}_2$). These subunits activate different signalling pathways. Along with the hydrolysis of *GTP*, another reaction partner *M* (e.g. arrestin) emerges (reaction ${\mathcal {R}}_3$), which initiates internalisation of the receptor–ligand complex (reaction ${\mathcal {R}}_4$). The $\beta \gamma$-subunit inhibits a membrane calcium channel by binding to it (reaction ${\mathcal {R}}_5$) (In other papers^20^ this is referred to as switching the calcium channel from the “willing” state to the “reluctant” state.). After dissociation of the $\beta \gamma$-subunit from the calcium channel, a trimeric G-protein complex is reformed, and the calcium channel is opened (reaction ${\mathcal {R}}_6$). The internalised receptor $RL_w$ is either recycled to the cell membrane (reaction ${\mathcal {R}}_7$) or degraded (reaction ${\mathcal {R}}_8$). The reaction partner *M* can itself be degraded (reaction ${\mathcal {R}}_9$). The ligand *L* can vanish before it binds to the receptor, e.g. by degradation or unspecific binding to other extracellular components (reaction ${\mathcal {R}}_{10}$), or it is degraded intracellularly (reactions ${\mathcal {R}}_7$ and ${\mathcal {R}}_8$). The constitutive G-protein activation is given by reaction ${\mathcal {R}}_{11}$, where we simply use ${\mathcal {R}}_2$ without ligand. That is, we model the constitutive binding reaction as a net reaction consisting of first the switching of GTP and GDP, followed by the dissociation of the $\beta \gamma$-subunit from the GPCR complex.

**Table 1:** Reactions and propensity functions.

*j*	Reaction ${\mathcal {R}}_j$	Propensity $f_j$
1	$L+R{\mathop {\longrightarrow }\limits ^{k_1}} RL$	$k_1\cdot x_R\cdot x_L$
2	$RL+\alpha _{GDP}\beta \gamma {\mathop {\longrightarrow }\limits ^{k_2}}RL+\alpha _{GTP}+\beta \gamma$	$k_2\cdot x_{RL}\cdot x_{\alpha _{GDP}\beta \gamma }$
3	$\alpha _{GTP}{\mathop {\longrightarrow }\limits ^{k_3}}\alpha _{GDP}+M$	$k_3\cdot x_{\alpha _{GTP}}$
4	$RL+M{\mathop {\longrightarrow }\limits ^{k_4}}RL_w$	$k_4\cdot x_{RL}\cdot x_{M}$
5	$\beta \gamma +Ca_{on}{\mathop {\longrightarrow }\limits ^{k_5}}Ca_{off}$	$k_5\cdot x_{ \beta \gamma }\cdot x_{Ca_{on}}$
6	$\alpha _{GDP}+Ca_{off}{\mathop {\longrightarrow }\limits ^{k_6}}\alpha _{GDP}\beta \gamma +Ca_{on}$	$k_6\cdot x_{\alpha _{GDP}}\cdot x_{Ca_{off}}$
7	$RL_w{\mathop {\longrightarrow }\limits ^{k_7}} R$	$k_7\cdot x_{RL_w}$
8	$RL_w{\mathop {\longrightarrow }\limits ^{k_8}}\emptyset$	$k_8\cdot x_{RL_w}$
9	$M{\mathop {\longrightarrow }\limits ^{k_9}}\emptyset$	$k_9\cdot x_{M}$
10	$L {\mathop {\longrightarrow }\limits ^{k_{10}}}$ $\emptyset$	$k_{10}\cdot x_{L}$
11	$R+\alpha _{GDP}\beta \gamma {\mathop {\longrightarrow }\limits ^{k_{11}}}R+\alpha _{GTP}+\beta \gamma$	$k_{11}\cdot x_{R}\cdot x_{\alpha _{GDP}\beta \gamma }$

For each reaction ${\mathcal {R}}_j$ there is a propensity function $f_j$ giving the rate (probability per unit of time) for the reaction to occur depending on the system’s state ${\varvec{x}}=(x_L,x_R,\ldots )$. For any species *S* it stands $x_S$ for the number of molecules of this species. *R:* receptor, *L:* ligand, *RL:* receptor–ligand complex, $RL_w$: internalised receptor, $\alpha _{GDP}\beta \gamma$ : G-protein, $\alpha _{GDP}/\alpha _{GTP}$: $\alpha$-subunit loaded with *GTP* or *GDP*, respectively, $\beta \gamma$: $\beta \gamma$-subunit, *M* reaction partner (e.g. arrestin) to initiate receptor internalisation, $Ca_{off}/Ca_{on}$: closed/open calcium channel.

Given these reactions, the stochastic dynamics of the system are mathematically modelled by a reaction jump process characterised by the chemical master equation^21,22^. The state of the system is given by a vector

$$\begin{aligned} {\varvec{x}}=(x_L,x_R,x_{RL},\ldots ) \in {\mathbb {N}}_0^{11} \end{aligned}$$

counting the number $x_S$ of molecules of the different species $S\in {\mathcal {S}}$, where ${\mathcal {S}}$ is the set of species under consideration:

$$\begin{aligned} {\mathcal {S}}:=\left\{ L,R,RL,RL_w,\alpha _{GDP}\beta \gamma , \alpha _{GDP}, \alpha _{GTP}, \beta \gamma , M, Ca_{on}, Ca_{off}\right\} . \end{aligned}$$

For each reaction ${\mathcal {R}}_j$ there is a stoichiometric vector $\varvec{\nu }_j\in {\mathbb {Z}}^{11}$ defining the net change in the population state ${\varvec{x}}$ induced by this reaction. That is, each time that reaction ${\mathcal {R}}_j$ occurs, this leads to a jump in the system’s state of the form

$$\begin{aligned} {\varvec{x}} \mapsto {\varvec{x}} + \varvec{\nu }_j. \end{aligned}$$

E.g., the stoichiometric vector $\varvec{\nu }_1$ of reaction ${\mathcal {R}}_1$ is given by $\varvec{\nu }_1=(-1,-1,1,0,\ldots ,0)$. The rates at which the reactions occur are given by propensity functions $f_j:{\mathbb {N}}_0^{11} \rightarrow [0,\infty )$, which can be found in the right column of Table 1.

The temporal evolution of the system is described by the Markov jump process $({\varvec{X}}(t))_{t\ge 0}$, ${\varvec{X}}(t)=(X_S(t))_{S\in {\mathcal {S}}}$, where $X_S(t)$ is the number of molecules of species *S* at time *t*. We define the probability $p({\varvec{x}},t):={\mathbb {P}}({\varvec{X}}(t)={\varvec{x}}|{\varvec{X}}(0)={\varvec{x}}_0)$ to find the system in state ${\varvec{x}}$ at time *t* given some initial state ${\varvec{x}}_0$. Then, the overall dynamics are characterised by the standard chemical master equation given by

1 $$\begin{aligned} \frac{d}{dt} p({\varvec{x}},t) = \sum _{j=1}^{11} \left[ f_j({\varvec{x}}-\varvec{\nu }_j)p({\varvec{x}}-\varvec{\nu }_j,t) - f_j({\varvec{x}})p({\varvec{x}},t) \right] . \end{aligned}$$

The reaction rate equation characterising the corresponding deterministic reaction system is given by the ordinary differential equation (ODE)

2 $$\begin{aligned} \frac{d}{dt}{\varvec{C}}(t) = \sum _{j=1}^{11}f_j({\varvec{C}}(t)) \varvec{\nu }_j \end{aligned}$$

for concentrations ${\varvec{C}}(t)=\frac{1}{V}{\varvec{X}}(t)$, where *V* is the system’s volume. It is well-known that the volume-rescaled Markov jump process $({\varvec{X}}(t)/V)_{0\le t\le T}$ governed by the chemical master equation (1) converges to the solution ${\varvec{C}}(t)_{0\le t\le T}$ of the ODE system (2) in the limit of large particle numbers, i.e., for $V\rightarrow \infty$^22^.

#### Stochastic vs deterministic approach

The stochastic approach has several advantages over the deterministic one. At first, ODEs are an approximation assuming that the higher moments are trivially given by powers of the first moment. Stochastic modelling is exact in the sense that it takes into account all higher moments. Furthermore, the stochastic approach is closer to reality because it assumes a finite set (discrete number) of molecules, while ODEs consider concentrations and only work as approximations for large particle numbers. So the stochastic model is better suited for modelling a small compartment like an axon terminal with a small number of MORs and G-proteins. For our analysis, we will consider comparatively small numbers of molecules for all species (concretely, 20 MORs and 40 G-proteins, see Table 3), such that the stochastic approach is indispensable. Last but not least, a stochastic model delivers more information than ODEs. E.g., it enabled us to analyse the variances of the trajectories or the probability distribution of certain variables like the number of ligand–receptor binding events, which will be done in “Isolated impact of pH value”.

In many situations, however, the ODE model provides a valid approximation of the rescaled first moment of the stochastic process, ${\varvec{C}}(t)\approx {\mathbb {E}}({\varvec{X}}(t))/V$, as it is also the case here. This fact will be exploited in “Parameter estimation” where the less complex ODE model instead of the stochastic one will be used for estimating the reaction rates $k_1,\ldots ,k_{10}$ based on experimental data.

### Laboratory in vitro experiments

**Figure 2:**
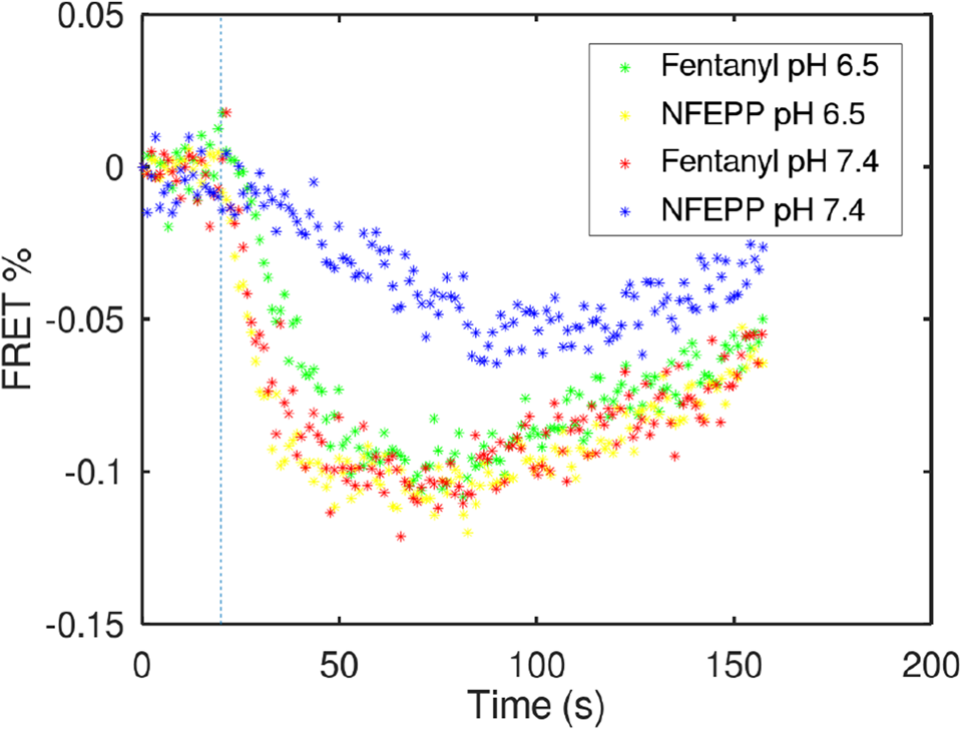
In vitro experiments: G-protein activation. Time course of ligand-induced G-protein subunit dissociation measured by FRET in HEK293 cells. FRET efficiency is depicted as percentage of initial intensities, corrected for photobleaching^3^. A higher number of dissociated G-protein subunits (stronger G-protein activation) is represented by more negative values. One can directly see that the blue “curve” (NFEPP at pH 7.4) shows lower numbers of dissociated subunits (weaker G-protein activation) compared to the other scenarios. The dashed line indicates the time point $t=20$ s where the ligand was added.

In order to validate our model, we performed laboratory experiments measuring G-protein activation and membrane calcium currents in vitro. To determine initial G-protein activation (as reflected by the exchange rate of GDP for GTP), the [$^{35}$S]-GTP$\gamma$S binding assay was used. Because these experiments require genetic alteration (by transfection) of cells, we performed these measurements in commonly used human embryonic kidney (HEK293) cells. In addition, we extracted data produced by FRET experiments^3^. These experiments measure ligand-induced G-protein subunit dissociation (which follows G-protein activation). The FRET experiments were used to fit the reaction rates. To mimic the mechanisms underlying in vivo opioid analgesia, we examined calcium currents in sensory neurons harvested from rodents using a patch-clamp protocol (see Supplementary Information for methodological details). The experimental results are shown in Figs. 2 and 5a below, and described in more detail in “Isolated impact of pH value”.

### Parameter estimation

Our model includes eleven previously unknown parameters $k_1,\ldots ,k_{11}$. The determination of appropriate values for these parameters included two main steps: a rough selection of values based on literature, followed by more precise standard parameter estimation.

In Ray et al.^7^, it has been shown by means of molecular dynamics (MD) simulations that the ligand binding affinity varies for different ligands and pH values. The protonation of both the ligand and certain residues in the receptor are important determinants for receptor activation, likely due to the pH-dependent formation of hydrogen bonds and/or salt bridges between ligand and receptor^18,19^. We used the relative changes of the rate constant as determined in Ray et al.^7^ and chose the following different values of $k_1$ for different ligand/pH combinations: $k_1=1.25\times 10^{-2}\,\text {s}^{-1}$ for fentanyl and pH 6.5, $k_1=2.5\times 10^{-2}\,\text {s}^{-1}$ for fentanyl/pH 7.4, $k_1=2.5\times 10^{-3}\,\text {s}^{-1}$ for NFEPP/pH 6.5, and $k_1=5\times 10^{-4}\,\text {s}^{-1}$ for NFEPP/pH 7.4. As the in vitro experiments used for the parameter estimation were performed in the absence of radicals, the rate $k_{11}$, which is responsible for the constitutive G-protein activation ${\mathcal {R}}_{11}$ , was set to zero, because constitutive activation probably does not play an important role in healthy tissue.

The parameters $k_2,\ldots ,k_{10}$ of the other intracellular reactions were assumed to depend only mildly (if at all) on the ligand/pH combination in the cellular environment; for each of these parameters a single value has been chosen, independent of ligand and pH. This is a reasonable assumption because we chose an intracellular pH value of 7.4 based on well-known mechanisms of cellular homeostasis: although transient (several minutes) changes of intracellular pH may occur with tissue acidosis, intracellular buffer systems and ion pumps in the plasma membrane will rapidly restore physiological pH to ensure cell viability^23^. Since most previous studies examined situations of longer-lasting inflammation (up to several days)^3–6^, we look at this situation, as well. Using results from Zamponi et al.^24^, we started by setting the rate constant $k_5$ of the central binding reaction between the $\beta \gamma$-subunit and the calcium channel to $k_5=5\times 10^{-2}\,\text {s}^{-1}$ and proceeded to arrange the other values relative to it according to what is known in the literature. Comparing previous work^24,25^ it can be deduced that ${\mathcal {R}}_5$ happens at a timescale an order of magnitude shorter than ${\mathcal {R}}_2$ and ${\mathcal {R}}_3$ (with ${\mathcal {R}}_2$ being slightly faster than ${\mathcal {R}}_3$), so we chose $k_2$ and $k_3$ five resp. ten times smaller than $k_5$. The rate constants of reactions ${\mathcal {R}}_4,{\mathcal {R}}_6,{\mathcal {R}}_9$ were assumed to be of the same magnitude as those of ${\mathcal {R}}_2$ and ${\mathcal {R}}_3$ (with ${\mathcal {R}}_6$ being slightly faster). The recycling and degradation of internalised ligand–receptor complexes are much slower, at a level of minutes (Fig. 1 in Williams et al.^26^) which leads to comparatively small rate constants $k_7$ and $k_8$ for the reactions ${\mathcal {R}}_7$ and ${\mathcal {R}}_8$ of the internalised receptor. The extracellular decay of ligand due to unspecific binding and other incidents (reaction ${\mathcal {R}}_{10}$) was set to a value at which it showed a first effect on calcium channel inhibition.

After this initial step of selecting rough parameter values based on available information, we fine-tuned the parameters $k_1,\ldots ,k_{10}$ via standard parameter estimation techniques using the in vitro experimental FRET data (see Fig. 2 below) that consists of four individual time series for G-protein activation for the four cases fentanyl/pH 6.5, fentanyl/pH 7.4, NFEPP/pH 6.5, and NFEPP/pH 7.4. The experimental data was first pre-processed by determining the offset time (time point at which the respective ligand was added), and the linear scaling transformation that maps the number of undissociated G-proteins from the model to the measured FRET signal. Then the residual distance between the solution of the ODE model and the experimental data was minimized by optimally adapting the parameters $k_2,\ldots ,k_{10}$, starting from the initial values previously chosen (second column of Table 2 termed “Preselected”). Here, the residual is the mean squared distance between ODE solution and data, summed over all four time series. The minimization was done using standard techniques for parameter estimation^27,28^, within the framework of the software PREDICI^29^. The resulting parameters values are shown in Table 2, third column termed “Parameter Estimation”. These values of $k_2,\ldots ,k_{10}$, optimally adapted to all four cases of ligand/pH combination at once, were fixed.

**Figure 3:**
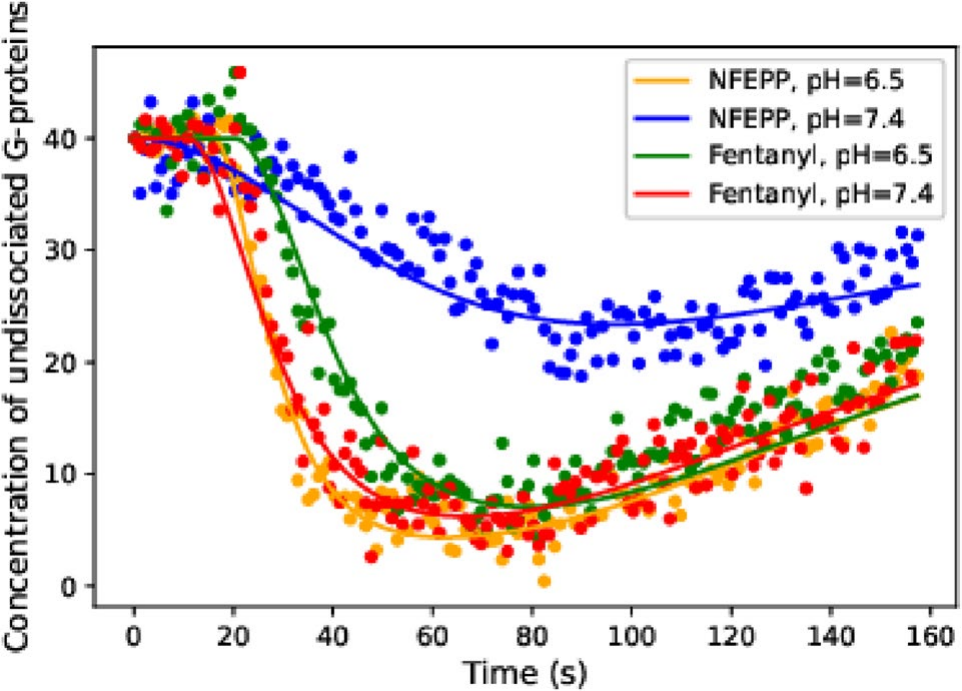
Experimental in vitro data and optimally fitted ODE model. Dots represent the time course of ligand-induced G-protein subunit dissociation measured by FRET in HEK293 cells. FRET values were transformed into concentration of undissociated G-proteins by a scaling factor. Lines indicate the best-fit of the ODE model to the data (using optimal parameters). For methodological details, see Spahn et al.^3^.

**Figure 4:**
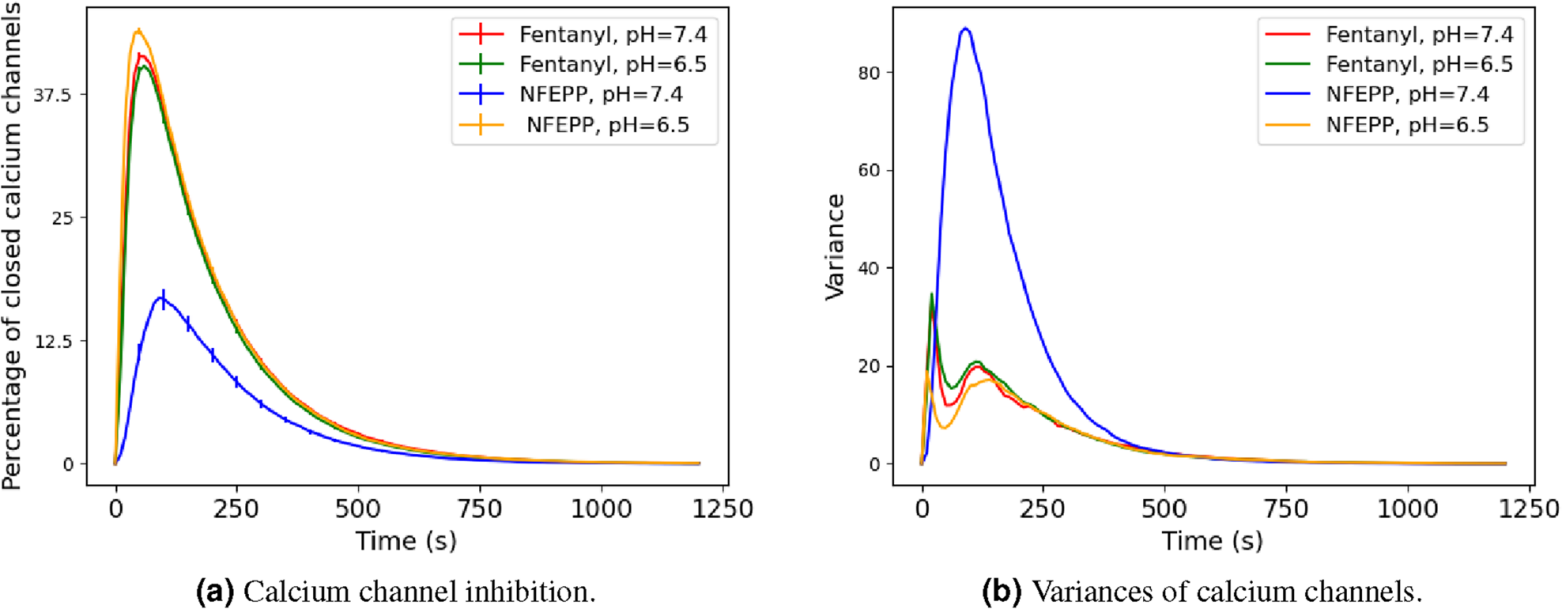
Numerical studies of the reaction process. (**a**) Time course of percentage of closed calcium channels for different values of the ligand-binding rate $k_1$. Error bars indicate 95%-confidence intervals (from 500 simulation runs). (**b**) Variances of closed calcium channels from 500 simulation runs.

**Figure 5:**
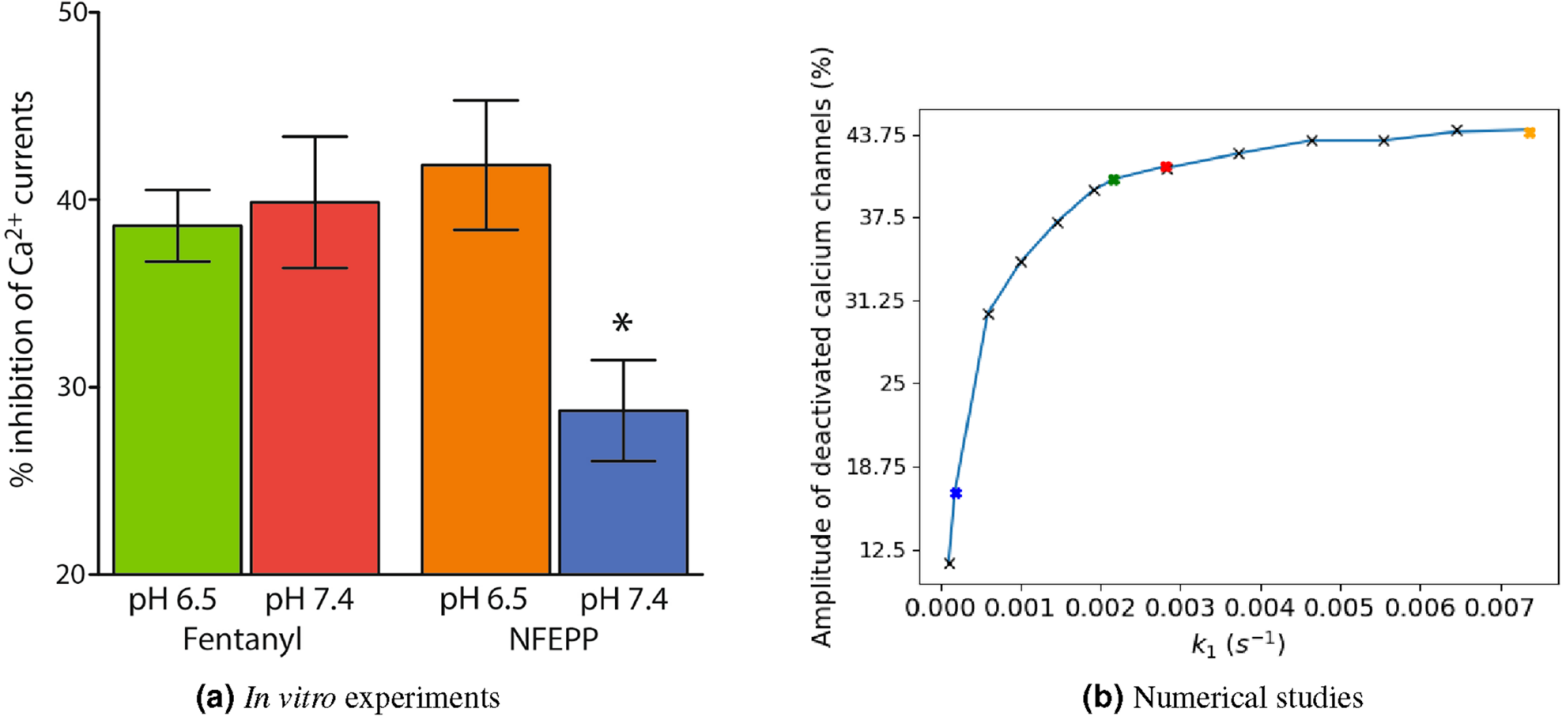
In vitro and numerical experiments: calcium currents. (**a**) Maximum inhibition of voltage-induced calcium currents by fentanyl or NFEPP at pH 6.5 and pH 7.4 measured by patch clamp experiments in rat sensory neurons. *$P < 0.05$ for comparison of NFEPP at pH 7.4 to all other values (one-way ANOVA with Bonferroni’s multiple comparisons). Data are means ± standard error of the mean (SEM). (**b**) Amplitude of the mean calcium channel inhibition plotted against $k_1$. Coloured crosses indicate the $k_1$ values according to Eq. (3).

In a final step, individually for each ligand/pH combination, the parameter $k_1$ was fine-tuned by minimizing the residual function for each single time-series for fixed $k_2,\ldots ,k_{10}$ by changing the respective $k_1$. The outcome was:3where colors shall help to identify the respective curves in Figs. 3, 4 and 5a.

Some of the optimal parameter values exhibit mild deviations from the preselected ones, but no stark contrast to the literature was observed; in fact, closer inspection showed that the mean squared deviation between model-based simulation and experiment was significantly reduced by fine-tuning parameters. The resulting best fit is shown in Fig. 3.

**Table 2:** Reaction rate constants.

Parameter	Preselected (in $\text {s}^{-1}$)	Parameter estimation (in s$^{-1}$)
$k_2$	$1.0 \times 10^{-2}$	$1.3\times 10^{-2}$
$k_3$	$5.0\times 10^{-3}$	$6.4\times 10^{-3}$
$k_4$	$5.0\times 10^{-3}$	$1.1\times 10^{-2}$
$k_5$	$5.0\times 10^{-2}$	$5.2\times 10^{-2}$
$k_6$	$2.5\times 10^{-2}$	$1.0\times 10^{-1}$
$k_7$	$5.0\times 10^{-4}$	$5.0\times 10^{-4}$
$k_8$	$5.0\times 10^{-5}$	$5.0\times 10^{-5}$
$k_9$	$5.0\times 10^{-3}$	$4.7\times 10^{-3}$
$k_{10}$	$2.5\times 10^{-2}$	$1.9\times 10^{-2}$

Optimal parameter values compared to the assumed values. The parameter $k_{11}$ is not listed here because its value is assumed to be fixed to $k_{11}=0$ for the parameter estimation. For $k_1$ see text.

It has been tested that, for these parameter values, the residual distance between data and stochastic model is very close to the residual distance for the ODE model.

### Numerical simulations of the reaction network

Simulations of the stochastic reaction network were performed using Python 3. For each combination of rate constants, 500 Monte Carlo simulations were carried out and the arithmetic mean was calculated in order to estimate the percentage of closed calcium channels plotted in Figs. 4 and  8. The initial state numbers of receptors, G-proteins and calcium channels were chosen at a ratio of 1:2:4 (see Table 3). These numbers are only a rough estimate since the exact stoichiometry of binding events in relation to the number of activated second messengers is currently not fully understood at the experimental level^30,31^. However, these numbers should suffice to get some first impressions of the properties of the reaction network.

As a time horizon for each simulation 1200 s were chosen. The results are presented in “Results”. In order to find the steady state of the dynamics under pure constitutive activation (i.e., ignoring the ligand-induced activation of receptors given by reaction ${\mathcal {R}}_1$), a simulation without ligands (or, equivalently, with $k_1=0$) was run. Given a non-zero rate constant $k_{11}$ for constitutive G-protein activation, we determined the long-term average number *a* of closed calcium channels under these conditions. This long-term mean *a* (rounded to natural numbers) of closed calcium channels was then used to determine the initial state for the dynamics including receptor activation (see Table 3 for the results). To check for normal distribution of the mean, the 500 runs were divided into batches of 50 and the respective means then tested. Anderson–Darling test indicated normal distribution with $P\le 0.05$, so the 95%-confidence interval of the t-distribution is shown in the plots.

**Table 3:** Initial state of the reaction process.

Species *S*	*L*	*R*	*RL*	$RL_w$	$\alpha \beta \gamma$	$\alpha _{GDP}$	$\alpha _{GTP}$	$\beta \gamma$	*M*	$Ca_{on}$	$Ca_{off}$
$X_S(0)$	10	20	0	0	$40-a$	*a*	0	0	0	$80-a$	*a*

Initial number $X_S(0)$ of molecules for each species $S\in {\mathcal {S}}$ used for all reaction network simulations, where $a=0$ for $k_{11}=0$ (“Isolated impact of pH value”) and $a=5$ for $k_{11}=5\times 10^{-5}$ (“Combined impact of pH value and increased radical concentration”).

## Results

We already demonstrated that the reaction network model introduced in “Models and methods” allows to explain the time course of G-protein subunit dissociation correctly for different ligands and pH values. Based on this validation step, the model was used to analyse the impact (a) of different extracellular pH values correlating to those occurring in injured tissues in vivo^2^ in combination with a conventional or a pH-dependent opioid ligand (fentanyl or NFEPP, respectively) (see “Isolated impact of pH value”), and (b) of radicals (see “Combined impact of pH value and increased radical concentration”) onto the overall signalling pathway. Based on our parameter fitting results, the changing pH value was modelled via varying the rate constant $k_1$ of ligand–receptor binding. Additional radicals, on the other hand, were assumed to lower the binding rate $k_1$ of opioids, while increasing the rate $k_{11}$ of constitutive activation^17,32^.

### Isolated impact of pH value

For the following analysis we set the rate constant $k_{11}$ to zero. Our goal was to analyse the effect of varying rates $k_1>0$ for the ligand-induced activation of a receptor (given by the binding reaction ${\mathcal {R}}_1: L+R \rightarrow RL$) onto the amount of closed calcium channels $Ca_{off}$. We examined the ligands fentanyl and NFEPP in combination with changing pH levels (see Eq. (3) for the respective rate values $k_1$). The rates for the other reactions were left unchanged in all reaction network simulations based on the assumption that intracellular pH remains at 7.4 (see Table 2).

Figure 4a represents the evolution of the mean number of closed calcium channels for the different ligand-binding rates $k_1$ given in Eq. (3). For all ligand and pH pairs except for NFEPP/pH 7.4 we observed similar amplitudes of closed calcium channels (about $44 \%$ of all calcium channels), while for NFEPP/pH 7.4 the amplitude is significantly reduced to approximately $29\%$ (but note that only a maximum of 40 out of the total of 80 channels can be closed since there are only 40 G-proteins, so the maximum calcium channel inhibition is $50\%$). In Fig. 4b, the variances of the number of closed calcium channels depending on time is shown. We can observe that this variance is significantly larger for NFEPP at a normal pH value than for all other scenarios. This information will be used later to support the conclusion that the reduction of the calcium channel inhibition is not due to a uniform decline of the trajectories.

#### Results from in vitro experiments

The maximum inhibition of voltage-induced calcium currents by fentanyl or NFEPP at pH 6.5 and pH 7.4 was measured by patch-clamp experiments in rat sensory neurons. The results are comparable to the scenarios simulated in Fig. 4a in that both fentanyl and NFEPP potently inhibited calcium currents at low pH, whereas NFEPP was significantly less effective than fentanyl at normal pH (Fig. 5a). Figure 2 shows the experiment that was used for data fitting.

#### Non-linear behaviour and stochastic effects

Seeing that our model resembled the in vitro results quite well, we now sought to get more information about the dependence of the calcium channel inhibition amplitude and the binding rate $k_1$. Therefore we plotted both against each other; the results are shown in Fig. 5b. We see a non-linear behaviour of the calcium channel inhibition amplitude with a rather sharp drop for $k_1<2\times 10^{-3}\,\text {s}^{-1}$ where the calcium channel response declines quickly.

**Figure 6:**
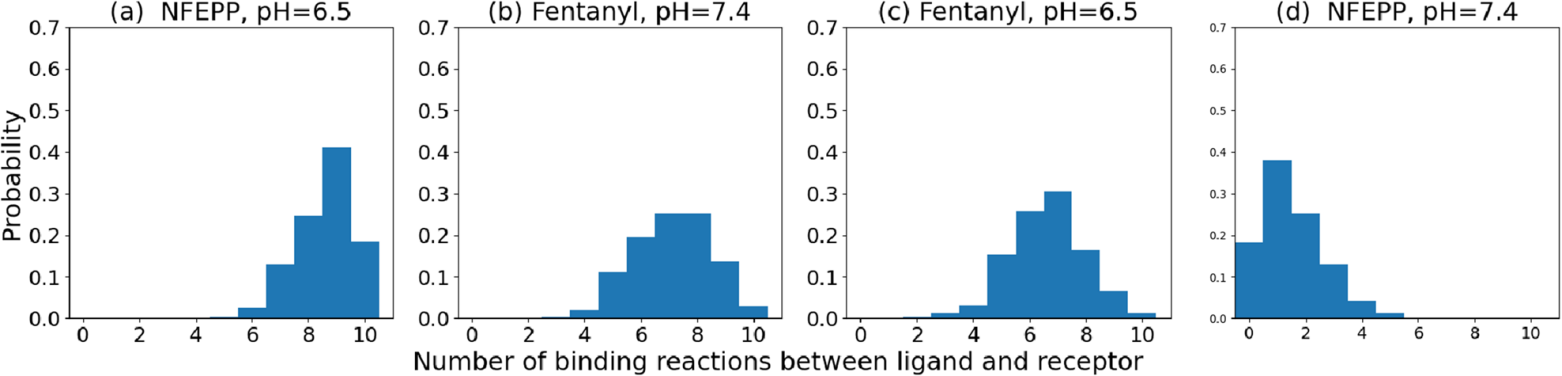
Numerical studies: number of reactions. Probability distribution of the number of receptor–ligand binding events ${\mathcal {R}}_1$ that happened during the whole time course of a simulation run (time interval $[0,1200\,\text {s}]$). For each $k_1$-level, 500 simulation runs have been evaluated.

In order to investigate the stochastic effects, we calculated the probability distributions of the number of binding events between ligands and receptors that happened during a simulation run over the time interval $[0,1200\,\text {s}]$ (see Fig. 6). For decreasing $k_1$ (see 3 for the corresponding ligand/pH pairs) the distribution shifts to lower values and gets a wider range. The non-linearity is also represented in the larger shift from (c) to (d) compared to the other shifts.

### Combined impact of pH value and increased radical concentration

We next investigated the effect of varying pH values in combination with rising radical levels on the signalling.

#### Results from in vitro experiments

**Figure 7:**
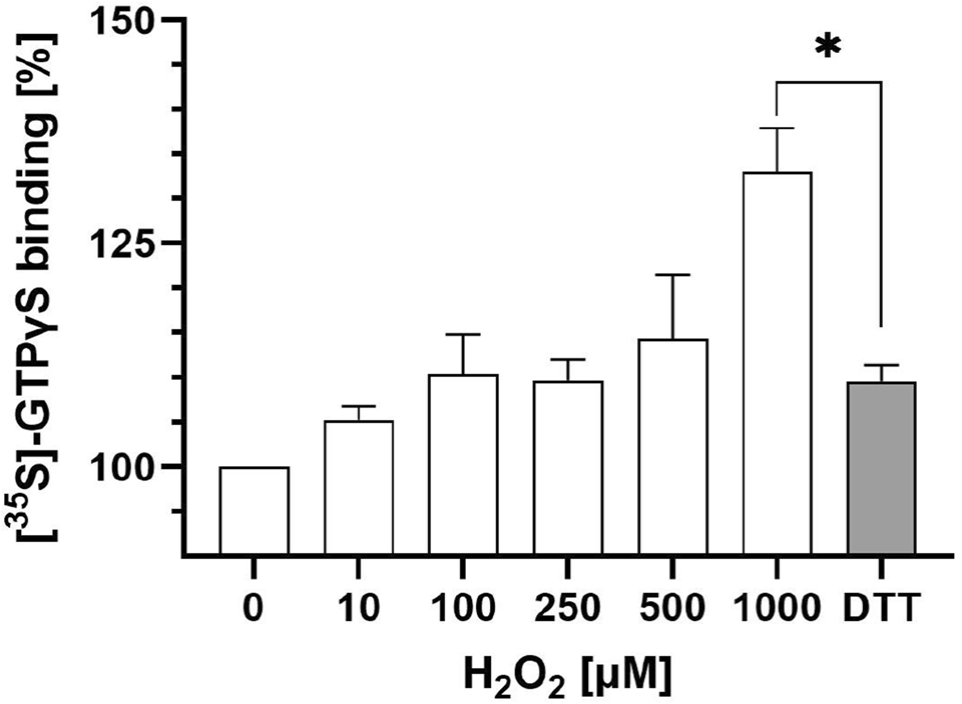
Experimental in vitro data on constitutive G-protein activation. Effects of increasing concentrations of radicals (induced by additional H$_{2}$O$_{2}$) without and with (last bar) DTT (5 mM) on basal [$^{35}$S]-GTP$\gamma$S binding to MOR without opioid ligands. Data are means ± SEM of specific binding normalised to the control group; $n=8$ per condition. $P < 0.05$, linear regression analysis. The asterisk indicates $P < 0.001$, t-test.

Reactive oxygen species can be produced in in vitro experiments by adding H$_{2}$O$_{2}$ to the sample (see Supplementary Information). Our experimental data support that increasing radical (H$_{2}$O$_{2}$) concentrations are correlated with increasing constitutive G-protein activation, see Fig. 7. This implies that we can include the possible influence of radicals by changing the rate $k_{11}$ for constitutive activation in the stochastic model.

According to the literature, increased radical concentrations promote the forming of disulfide bonds (DSB) in the receptor ^14,33^. Hypothetically, the constitutive activation observed in our in vitro experiments stems from DSB formation because adding dithiothreitol (DTT)—a reducing agent that disrupts DSB—reversed the effect of the highest concentration (1 mM) of H$_{2}$O$_{2}$ on basal G-protein activation (Fig. 7). More recent reports further support that DSB formation influences the functionality of GPCR^15,16^, and there is evidence that constitutive MOR activity is associated with enhanced accessibility of cysteine residues^34^ (which is essential for DSB formation).

The formation of DSB by radicals has also been investigated by other groups, considering the effect onto ligand-based activation of the GPCR (in our model referring to the binding rate $k_1$). For example, Zhang et al.^32^ describe decreased ligand binding after the removal of a DSB in the extracellular part of the MOR. A review article by Wheatley et al.^17^ mentions decreased agonist affinity at the CXC-chemokine receptor 4 and increased constitutive activity of the angiotensin II type 1 receptor after breaking extracellular DSBs. In all of the mentioned examples in literature, the change of the receptor structure due to a change of the pristine DSB constellation decreases the binding affinity of specifically designed ligands. Concerning our model, these results indicate that when studying inflamed tissue including increased radical concentrations, the values of the ligand activation rate $k_1$ should be reduced.

#### Changing the stochastic model accordingly

With progressive inflammation, there are now two effects on $k_1$, one from the pH and one from radicals. For an analysis of the combined effect, taking into account the effect of additional DSB formation, the values for $k_1$ given in Eq. (3) were reduced to $80\%$ for the inflamed scenario (pH 6.5, additional radicals). For the concrete values see Table 4.

As for the constitutive activation, our in vitro experiments imply that we can include the influence of additional radicals by increasing the corresponding rate constant $k_{11}$. The experimental data available for the scenario including radicals do not allow for a direct parameter estimation of $k_{11}$. We chose the value $k_{11}=5 \times 10^{-5}$ in the presence of radicals, inducing a base level of approximately 5 closed calcium channels in the healthy tissue scenario, which appears to be a reasonable value compared to the other model parameter values. In the scenario without radicals, we set $k_{11}=0$ as before.

**Figure 8:**
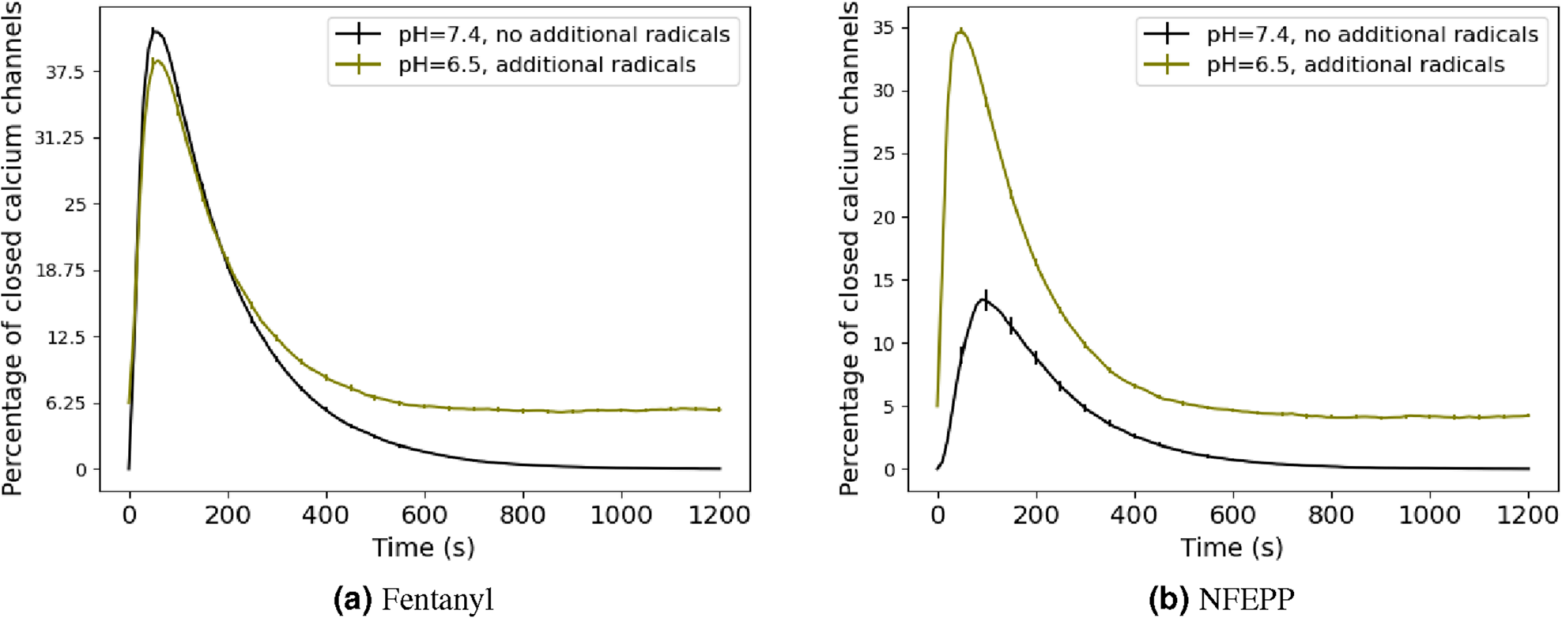
Numerical studies of calcium channel deactivation. Time course of percentage of closed calcium channels for different values of the ligand-binding rate $k_1$ and the constitutive activation rate $k_{11}$ for the ligands (**a**) fentanyl and (**b**) NFEPP. We set $k_{11}=0$ for the case of no radicals and $k_{11}=5\times 10^{-5}$ in case of radicals presence, while the values of $k_1$ are given in Table 4. Black curves represent the healthy tissue situation, while olive shows the effects of more inflammation (lower pH, more radicals). The time axis is the same as in Fig. 4a. Error bars indicate 95%-confidence interval (for 500 simulation runs).

According to the resulting model, the base level of closed calcium channels increases with progressive inflammation (i.e. rising radical concentrations). This is seen in Fig. 8 which shows two plots, one for fentanyl and one for NFEPP. The black curve represents the healthy tissue situation (pH 7.4, no additional radicals) while the olive (pH 6.5, additional radicals) curve shows the effects of progressive inflammation. The main difference between NFEPP and fentanyl is the increase of the amplitude/peak value of the closed calcium channels for NFEPP in the healthy scenario, which is not altered by the constitutive receptor activity.

**Table 4:** Receptor–ligand binding rates.

Ligand	pH $=7.4$, no additional radicals	pH $=6.5$, additional radicals
Fentanyl	$2.81\times 10^{-3}\,\text {s}^{-1}$	${{1.73}}{{\times }}{10}^{{-3}}\,\text {s}^{{-1}}$
NFEPP	$1.79\times 10^{-4}\,\text {s}^{-1}$	${{6}}{{\times }}{10}^{{-3}}\,\text {s}^{{-1}}$

Rate constant $k_1$ for receptor activation by ligand-binding, depending on the ligand, the pH-level and radicals presence. These values result from the ones given in Eq. (3) after multiplying by the factor 0.8 for the inflamed scenario (pH 6.5, additional radicals) while leaving unchanged for pH $=7.4$ and no radicals.

## Discussion

We presented a stochastic model of a canonical GPCR signalling pathway linked to plasma membrane function. This pathway is composed of a biochemical reaction network which begins at the receptor, continues with the G-protein and extends to the membrane calcium channels. The respective reaction rates were determined by fitting the model to data from in vitro FRET experiments. In addition, we have studied the role of radicals.

The validated model shows a non-linear behaviour of the calcium channel inhibition response with regard to the change of the receptor–ligand binding rate $k_1$. A critical value of $k_1$ at which the response drops markedly, is $k_1=2\times 10^{-3}\,\text {s}^{-1}$ (see Fig. 5b). In the vicinity of this value, small changes of $k_1$ lead to large changes of calcium channel inhibition response. For the ligand NFEPP, decreasing pH from its level for healthy tissue to inflamed tissue lets $k_1$ increase from below to above this critical value, so that increasing pH results in a quickly decreasing effect of NFEPP. For fentanyl, this is not the case. That is, we observe a markedly diminished effect (i.e. a lower number of closed calcium channels) of NFEPP at normal pH compared to all other scenarios (NFEPP at low pH, fentanyl at low or normal pH).

These results support our previous studies demonstrating that the conventional ligand fentanyl activates MOR both in injured (low pH) and non-injured (normal pH) tissues, while NFEPP is not active in non-injured environments (brain, intestinal wall)^3–6^. Those findings were attributed to the decreased pKa value of NFEPP (pKa = 6.8). Apparently, the decreased pKa precludes the protonation of NFEPP in healthy tissues (pH > 7.35; e.g. in brain) but facilitates its protonation in injured/inflamed microenvironments (low pH, high concentrations of protons). Consistent with the notion that the protonation of opioid ligands is typically required for the binding to opioid receptors^19^, NFEPP was active only in injured tissues. In addition, protonation likely plays a significant role in the activation process of most GPCRs^35^.

We were able to investigate this phenomenon further: for NFEPP and normal pH, we see higher fluctuations around a lower average of closed calcium channels, compared to all other sceanrios (NFEPP at low pH, fentanyl at low or normal pH).

These results are an extension of the findings in our earlier work^3^. There it was theorised and corroborated in animal studies that a ligand with proper pH-dependent binding rate would exhibit analgesic effects without side effects. Now we can add that the change of binding rates results in reduced calcium channel inhibition. Thus, the present data provide a more detailed explanation by including the intracellular signalling pathway underlying our initial findings. This further supports our concept of targeting disease-specific conformations of MOR to preclude adverse side effects of painkillers.

Moreover, we included an analysis of constitutive MOR activation with increased concentration of radicals. Comparing the influence of two prominent inflammatory mediators (pH and radicals) on ligand-induced opioid receptor function, it seems that pH has a higher impact than radicals under the chosen parameters. When designing novel opioid painkillers devoid of side effects elicited in non-injured environments, pH-sensitivity may be more important than radical-sensitivity which mainly affects the “base line” and not the peak value in Fig. 8. Given the high degree of homology between GPCRs^1^, our current studies may be applicable to other signalling pathways (e.g. from receptor to nucleus^12^), to GPCR involved in other diseases (e.g. cancer, high blood pressure, addiction, depression, arthritis) or even to non-human GPCRs in deranged environments (e.g. in animals or plants exposed to ocean acidification^36–43^).

## Supplementary Information

Supplementary Information.

## Data Availability

The datasets generated during and/or analysed during the current study are available in the github repository, https://github.com/user3849/MOR1.
