# Supplementary material for: Modelling altered signalling of G-protein coupled receptors in inflamed environment to advance drug design
Source: Sci Rep. 2023 Jan 12;13:607. doi: 10.1038/s41598-023-27699-w (PMC9837128; doi:10.1038/s41598-023-27699-w)
Supplement: Supplementary file 1 — Supplementary Information. [file 41598_2023_27699_MOESM1_ESM.pdf]

Supplementary Information

by A. Thies, V. Sunkara, S. Ray, H. Wulkow, M. Özgür Celik, F. Yergöz,
C. Schütte, C. Stein, M. Weber, S. Winkelmann

In vitro experiments

Measurement of calcium currents in sensory neurons

To mimic the mechanisms underlying *in vivo* opioid analgesia, we examined calcium currents in sensory neurons harvested from rodents using a patch clamp protocol modified from[42]. The following chemicals were used: Dulbecco's Modified Eagles Medium (DMEM)/HAM's F-12 medium (Biochrom F4815, Berlin, Germany), Penicillin (10,000 U), Streptomycin (10 mg/ml), 1.25% Collagenase (Sigma-Aldrich C0130, Taufkirchen, Germany), 2.5% Trypsin (Sigma-Aldrich T0303), acridine orange/propidium iodide (Logos, Villeneuve, France), $CaCl_2 \cdot 6H_2O$, TEA-Cl_2, 4-(2-hydroxyethyl)-1-piperazineethanesulfonic acid (HEPES), d-glucose, CsCl, $MgCl_2$, ethylene glycol-bis-(β-aminoethyl ether)-N,N,N',N'-tetraacetic acid (EGTA), Mg-ATP, GTP (Sigma-Aldrich).

Dorsal root ganglia (DRG) were harvested from naïve male Wistar rats (200-300 g; Janvier, Le Genest-Saint-Isle, France). Rats were killed by an overdose of isoflurane (AbbVie, Wiesbaden, Germany). The thoracic and lumbar spinal regions were exposed, DRG were collected in a digestive solution with 1.25% collagenase and incubated for 60 min at 37°C. After washing the cells three times with phosphate buffered saline (PBS), they were incubated in a digestive solution with trypsin for another 10 min at 37°C. After digestion, the tissue was triturated using plastic pipette tips and subsequently filtered through a 40 μl filter. The filtrate was centrifuged, the supernatant was discarded and the pellet was resuspended in 1 ml culture medium (DMEM/HAM's F12 supplemented with 1% penicillin/streptomycin and 10% horse serum). Cells were then seeded onto poly-L-lysine coated plastic culture dishes (35 mm) and placed in an incubator (5% CO_2 at 37°C). One hour later, the cell cultures were topped up to a total of 2 ml of culture medium and cultured until patch clamp recordings, as previously described[43].

Recordings from DRG neurons were performed 24–48 h after plating. Cell viability was evaluated before the first experiment by an automated cell counter (Luna, Villeneuve, France) using acridine orange/propidium iodide. Recordings were carried out in whole-cell voltage clamp mode. After washing with PBS, cells were bathed in an extracellular buffer (ECS) (10 mM $CaCl_2 \cdot 6H_2O$, 130 mM TEA-Cl_2, 5 mM HEPES, 25 mM d-glucose; adjusted to pH 7.4 or 6.5; all from Sigma-Aldrich) and visualised using a Zeiss Axiovert 200 inverse microscope (Zeiss, Jena, Germany). Patch pipettes (resistance 3.5–8 MΩ) were produced from Borosilicate glass capillaries using a Sutter P-97 puller (Sutter Instruments, Novato, CA, USA) and filled with intracellular buffer (105 mM CsCl, 2.5 mM $MgCl_2$, 40 mM HEPES, 10 mM EGTA, 2 mM Mg-ATP, 0.5 mM GTP, 5 mM d-glucose; adjusted to pH 7.4 or 6.5; all from Sigma-Aldrich). Currents were amplified and recorded using an EPC-10 patch amplifier and Pulse software (HEKA, Lambrecht, Germany). Extracellular buffer was added in a steady flow of 800–1,000 μl/min using a pressurised application system (Perfusion Pressure Kit VPP-6; Warner Instruments, Hamden, CT, USA) and a suction pump. Opioid ligands (fentanyl, NFEEP, naloxone) were applied using a perfusion valve system (VC-6; Warner Instruments) to switch between vehicle (buffer) and the test compounds. After reaching the "giga-seal" at -60 mV, the membrane patch was breached to achieve whole-cell configuration. Only cells showing proper action potentials were selected for further experiments. The currents were initially recorded at a holding potential of -80 mV in ECS buffer in the absence of opioid ligands. Immediately thereafter, the cells were depolarised to +10 mV (100 ms) for eight times after 20 s intervals. During the first five cycles, only ECS was applied. On the sixth cycle, separate (independent) sensory neurons were briefly exposed to fentanyl or NFEPP at either pH 6.5 or pH 7.4, and the ligand was washed out thereafter. The number (n) of measurements was as follows: fentanyl (pH 6.5) $n = 24$; fentanyl (pH 7.4) $n = 18$; NFEPP (pH 6.5) $n = 17$; NFEPP (pH 7.4) $n = 15$. All recordings were performed at room temperature.

Measurement of G-protein activation

Because these experiments require genetic alteration (by transfection) of cells, we performed these measurements in commonly used human embryonic kidney (HEK293) cells (RRID:CVCL 0045, German Collection of microorganisms and Cell Cultures, Braunschweig, Germany). All chemicals were purchased from Sigma-Aldrich (Taufkirchen, Germany), unless otherwise stated. [^{35}S]-guanosine-5'-O-(3-thio)-triphosphate ([^{35}S]-GTPγS) was purchased from Perkin Elmer (Waltham, USA). Cell culture reagents were purchased from Biochrom (Berlin, Germany).

Cells were maintained in DMEM supplemented with fetal bovine serum (Biochrom), penicillin (100 U/ml, Biochrom) and streptomycin (100 μg/ml, Biochrom) with or without geneticin (G418, 100 μg/ml, Biochrom), in 5% CO_2 at 37 °C as described before[3]. Cells were passaged 1:3 - 1:20 every second to third day from p8 and p28 depending on confluence. Cells were plated on culture dishes coated with poly-L-lysine 24 h before transfection. 24 h after seeding, confluent cells (70-90%) were

transfected with 1 μg per 200 μl transfection mix of each plasmid containing the different cDNAs using X-tremeGENE HP DNA Transfection Reagent (Roche, Mannheim, Germany) following the manufacturer's instructions. For stable transfection, pcDNA^{TM}3.1+ carrying the rat MOR provided by Christian Zöllner (University Hamburg, Germany) was linearised with restriction enzyme Bg1II (NEB, Frankfurt, Germany), and linearisation was verified by agarose gel electrophoresis. After 48 h, the medium containing the transfection reagent was removed and replaced by complete DMEM with 10% fetal bovine serum and penicillin/streptomycin (100 U/ml). Successfully transfected cells were selected by adding G418 (500 μg/ml) into medium that was renewed every 2 to 3 days. Monoclonal cell lines were then created 17 days post transfection by picking single colonies of stably transfected cells using a 100 μl pipette and transferring them to poly-L-Lysine coated wells of a 96-well plate. Cells were grown to confluence and successively transferred to larger culture flasks in the continued presence of 500 μg/ml G418. Antibiotic concentration was reduced to 100 μg/ml when the cells were moved to 75 cm^2 culture flasks. Monoclonal cell lines were further characterised based on immunocytochemistry, MOR mRNA expression, subjective impression of cell growth and overall cell morphology, as described previously[3]. Stably transfected cell lines were cultured for a maximum of 23 passages.

Protein concentrations were determined with the Bradford assay using Coomassie Brilliant Blue G-250 dye (Bio-Rad Laboratories GmbH, München, Germany) that shifts absorption from 465 to 595 nm upon binding to proteins. The relationship between measured absorbance and protein concentration was established based on a standard curve obtained from fixed protein solutions of known composition and concentration. These measurements were performed in duplicates using Bio-Rad Protein Assay Dye Reagent Concentrate with Bio-Rad Protein Assay Standard II (Bio-Rad). Samples with unknown concentrations, standards and dye reagent concentrate were diluted according to the manufacturer's instructions, thoroughly mixed, and incubated for 5 min at room temperature. Absorption at 595 nm was measured in triplicates with a spectrophotometer. Generation of linear standard curves and interpolation of total protein concentration was performed by the device's inbuilt software. A standard curve was generated for every experiment.

Membrane fractions were prepared from transfected HEK293 cells as described previously[44]. The cells were grown in 175 cm^2 tissue culture flasks to approximately 90% confluence. Cells were then washed with Tris buffer (50 mM, Trizma preset crystals, pH 7.4; Sigma Aldrich), harvested with a scraper, homogenised using a mechanical disperser (Dispergierstation T8.10, IKA-Werke, Staufen, Germany) at maximum speed for 10 s and centrifuged at 42K$\times$g for 20 min at 4°C (Avanti JXN-26 ultracentrifuge, Beckmann Coulter, Krefeld, Germany). Cellular pellets including membranes with embedded and anchored proteins were then resuspendend in Tris buffer for washing to separate them from cytosolic components by homogenisation and centrifugation at the same settings. Supernatants were discarded and the pellets were stored at -80 °C. On the day of usage, the pellets were thawed on ice in Tris buffer and homogenised. Total protein concentrations were determined as described above and homogenates were split according to the number of conditions tested in respective assay buffers.

The [^{35}S]-GTPγS binding assay was used to determine G-protein activation (as reflected by the exchange rate of GDP for GTP) at different H_2O_2 concentrations (0-1,000 μM). GTP was replaced by a high concentration of [^{35}S]-GTPγS in the assay solvent, and the accumulation of [^{35}S]-GTPγS-bound G proteins in the membrane was measured. Membrane fractions were prepared with the following modifications: Membranes were homogenised and dissolved in HEM G-protein buffer containing 8 mM HEPES, 8 mM 4-(2-Hydroxyethyl)-1-piperazinepropanesulfonic acid (EPPS), 8 mM 2-(N-Morpholino)-ethanesulfonic acid (MES), 100 mM NaCl, 0.2 mM EGTA, 5 mM $MgCl_2$ at pH 7.6, including freshly added 0.1% (w/v) bovine serum albumin (BSA). The desired amount of H_2O_2 was then added. To avoid interference with reactive oxygen species, the reducing agent dithiothreitol (DTT) (as originally used in[44]) was omitted. Basal [^{35}S]-GTPγS binding was assessed in the presence of vehicle without opioid ligands. In analogy to[45], 50 μg of membrane fractions in duplicates were incubated with GDP (30 μM) and [^{35}S]-GTPγS (0.05 nM) for 90 min at 30 °C. To investigate reducing effects, separate samples pretreated with the highest concentration of H_2O_2 (1 mM) were incubated for another 30 min with DTT (5 mM) at 30 °C. Unspecific [^{35}S]-GTPγS binding in the presence of non-radioactive GTPγS (10 μM) was subtracted to yield specific binding. Bound and free ligands were separated by rapid filtration under vacuum through Whatman GF/B glass fiber filters soaked in water followed by 6 washes with Tris Buffer. Bound radioactivity was determined by liquid scintillation spectrophotometry for ^{35}S after overnight extraction of the filters in scintillation fluid optiphase HISAFE 3 (Perkin Elmer, Waltham, USA). Concentrations of radioactive compound were calculated based on the half life of ^{35}S (87.4 days). Experiments were randomised to compensate for position effects in the filter apparatus or unequal sample processing times. Data processing and analysis were blinded for different H_2O_2 concentrations with the help of a colleague.

The data on dissociation of G-protein subunits, as measured by FRET, were extracted from[3]. Methodological details are described in[3].

Data Analysis

Experimental designs were randomised to compensate for the position effects on plates or filter apparatus and unequal sample processing time. Sample sizes were calculated using the G*Power 3.1.2 program with $\alpha < 0.05$, a power of 80% and a defined effect size (derived from pilot experiments). Analysis of concentration-response relationship was performed with simple linear regression using the GraphPad Prism 9 program (GraphPad, San Diego, USA) where $y =$ [^{35}S]-GTPγS bound and $x =$

[H_2O_2]. A P value ≤ 0.05 was considered statistically significant. Normal distribution of the data was assessed using the Kolmogorov-Smirnov test. Data are represented as means $\pm$ standard error of the mean (SEM).

References

1. Congreve, M., de Graaf, C., Swain, N. A. and Tate, C. G. Impact of GPCR structures on drug discovery. *Cell*, **181(1)**:81–91, 2020.
2. Stein, C. New concepts in opioid analgesia. *Expert opinion on investigational drugs*, **27(10)**:765–775, 2018.
3. Spahn, V. et al. A nontoxic pain killer designed by modeling of pathological receptor conformations. *Science*, **355**:966–969, 2017.
4. Rodriguez-Gaztelumendi, A., Spahn, V., Labuz, D., Machelska, H. and Stein, C. Analgesic effects of a novel pH-dependent μ-opioid receptor agonist in models of neuropathic and abdominal pain. *Pain*, **159**:2277–84, 2018.
5. Massaly, N., Temp, J., Machelska, H. and Stein, C. Uncovering the analgesic effects of a pH-dependent μ-opioid receptor agonist using a model of non-evoked ongoing pain. *Pain*, **161**:2798–804, 2020.
6. Jimenez-Vargas, N. N. et al. Agonist that activates the μ-opioid receptor in acidified microenvironments inhibits colitis pain without side effects. *Gut*, Epub ahead of print, 2021.
7. Ray, S., Sunkara, V., Schütte, C. and Weber, M. How to calculate pH-dependent binding rates for receptor-ligand systems based on thermodynamic simulations with different binding motifs. *Molecular Simulation*, **46(18)**:1443 – 1452, 2020.
8. Bhave, G. and Gereau, R. W. Posttranslational mechanisms of peripheral sensitization. *Journal of Neurobiology*, **61**:88–106, 2004.
9. Weis, W. I. and Kobilka, B. K. The molecular basis of GPCR activation. *Annu. Rev. Biochem.*, **87**:897–919, 2018.
10. Proft, J. and Weiss, N. G-protein regulation of neuronal calcium channels: Back to the future. *Mol Pharmacol*, **87**:890–906, 2015.
11. Zamponi, G. W., Striessnig, J., Koschak, A. and Dolphin, A. C. The physiology, pathology, and pharmacology of voltage-gated calcium channels and their future therapeutic potential. *Pharmacological Reviews*, **67**:821–870, 2015.
12. Shaw, W. M. et al. Engineering a model cell for rational tuning of GPCR signaling. *Cell*, **177(3)**:782–796, 2019.
13. Bridge, L. J., Mead, J., Frattini, E., Winfield, I. and Ladds, G. Modelling and simulation of biased agonism dynamics at a G-protein-coupled receptor. *Journal of theoretical biology*, **442**:44–65, 2018.
14. Bowen, W. D. and Pert, C. B. Conformational malleability of opiate receptors: sulfhydryl modification alters ion-induced μ/δ-ligand selectivity shifts in rat striatal sections. *Cellular and Molecular Neurobiology*, **2**:115–128, 1982.
15. Yang, F. et al. Structure, function and pharmacology of human itch receptor complexes. *Nature*, **600(7887)**:164–169, 2021.
16. Cao, C. et al. Structure, function and pharmacology of human itch GPCRs. *Nature*, **600(7887)**:170–175, 2021.
17. Wheatley, M. et al. Lifting the lid on GPCRs: the role of extracellular loops. *British Journal of Pharmacology*, **165(6)**:1688–1703, 2012.
18. Currie, K.P.M. G-protein inhibition of cav2 calcium channels. *Channels*, **4:6**:497–509, 2010.
19. Gillespie, D. T. A rigorous derivation of the chemical master equation. *Physica A: Statistical Mechanics and its Applications*, **188(1-3)**:404–425, 1992.
20. Winkelmann, S. and Schütte, C. *Stochastic Dynamics in Computational Biology*. Springer, 2020.
21. Boron, F. W. Regulation of intracellular pH. *Advances in Physiology Education*, **28**:160–169, 2004.
22. Zamponi, G. W. and Snutch, T. P. Decay of prepulse facilitation of N type calcium channels during G-protein inhibition is consistent with binding of a single $g_{\beta\gamma}$ subunit. *Proc. Natl. Acad. Sci. USA*, **95**:4035–4039, 1998.
23. Shea, L. D., Neubig, R. R. and Linderman, J. J. Timing is everything the role of kinetics in G-protein activation. *Life Sciences*, **68**:647–658, 2000.
24. Williams J. T. et al. Regulation of μ-opioid receptors: Desensitization, phosphorylation, internalization, and tolerance. *Pharmacological Reviews*, **65**:223–254, 2013.
25. Bock, H. et al. *Model based parameter estimation. Theory and applications. Based on the workshop on parameter estimation*, volume 4. Springer, 2013.

26. Wulkow, N., Telgmann, R., Hungenberg, K.-D., Schütte, C. and Wulkow, M. Deterministic and stochastic parameter estimation for polymer reaction kinetics I: Theory and simple examples. *Macromolecular Theory and Simulations*, 2021.

27. Wulkow, M. Computer aided modeling of polymer reaction engineering—the status of predici, I-simulation. *Macromolecular Reaction Engineering*, **2(6)**:461–494, 2008.

28. Hilger, D. The role of structural dynamics in GPCR-mediated signaling. *FEBS Journal*, **288(8)**:2461–2489, 2021.

29. Latorraca, N. R., Venkatakrishnan, A. J. and Dror, R. O. GPCR dynamics: Structures in motion. *Chemical Reviews*, **117(1)**:139–155, 2017.

30. Cremers, C. M. and Jakob, U. Oxidant sensing by reversible disulfide bond formation. *Journal of Biological Chemistry*, **288(37)**:26489–26496, 2013.

31. wwPDB consortium. Protein data bank: the single global archive for 3d macromolecular structure data. *Nucleic Acids Research*, **47(D1)**:D520–D528, 2019.

32. Koehl, A. et al. Structure of the μ-opioid receptor-G-protein complex. *Nature*, **558(7711)**:547–552, 2018.

33. Isberg, V. et al. Generic GPCR residue numbers – aligning topology maps while minding the gaps. *Trends in Pharmacological Sciences*, **36(1)**:22–31, 2015.

34. Li, J.-G. et al. Asp147 in the third transmembrane helix of the rat μ-opioid receptor forms ion-pairing with morphine and naltrexone. *Life Sciences*, **65(2)**:175–185, 1999.

35. Mahinthichaichan, P., Vo, Q. N., Ellis, C. R. and Shen, J. Kinetics and mechanism of fentanyl dissociation from the μ-opioid receptor. *JACS Au*, **1(12)**:2208–2215, 2021.

36. Meyer, J., Del Vecchio, G., Seitz, V., Massaly, N. and Stein, C. Modulation of μ-opioid receptor activation by acidic pH is dependent on ligand structure and an ionizable amino acid residue. *British Journal of Pharmacology*, **176(23)**:4510–4520, 2019.

37. Zhang, H., Kenaan, C., Hamdane, D., Hoa, G. H. B. and Hollenberg, P. F. Effect of conformational dynamics on substrate recognition and specificity as probed by the introduction of a de novo disulfide bond into cytochrome p450 2b1. *Journal of Biological Chemistry*, **284(38)**:25678–25686, 2009.

38. Dror, R. O. et al. Activation mechanism of the β2-adrenergic receptor. *Proceedings of the National Academy of Sciences*, **108(46)**:18684–18689, 2011.

39. Fowler, C. B., Pogozheva, I. D., Lomize, A. L., LeVine, H. III and Mosberg, H. I. Complex of an active μ-opioid receptor with a cyclic peptide agonist modeled from experimental constraints. *Biochemistry*, **43(50)**:15796–15810, 2004.

40. Zhang, P. et al. Mutation of human μ-opioid receptor extracellular "disulfide cysteine" residues alters ligand binding but does not prevent receptor targeting to the cell plasma membrane. *Molecular Brain Research*, **72(2)**:195–204, 1999.

41. Xu, W., Sanz, A., Pardo, L. and Liu-Chen, L.-Y. Activation of the μ-opioid receptor involves conformational rearrangements of multiple transmembrane domains. *Biochemistry*, **47(40)**:10576–10586, 2008.

42. Walwyn, W., Evans, C. J. and Hales, T. G. β-Arrestin2 and c-Src regulate the constitutive activity and recycling of μ-opioid receptors in dorsal root ganglion neurons. *Journal of Neuroscience*, **27(19)**:5092–5104, 2007.

43. Nockemann, D. et al. The K^+ channel GIRK2 is both necessary and sufficient for peripheral opioid-mediated analgesia. *EMBO Molecular Medicine*, **5**:1263–1277, 2013.

44. Zöllner, C. et al. Painful inflammation-induced increase in μ-opioid receptor binding and G-protein coupling in primary afferent neurons. *Molecular Pharmacology*, **64(2)**:202–210, 2003.

45. Ludwig, M-G. et al. Proton-sensing G-protein-coupled receptors. *Nature*, **425(6953)**:93–98, 2003.

46. Zhang, X. C., Cao, C., Zhou, Y. and Zhao, Y.. Proton transfer-mediated GPCR activation. *Protein Cell*, **6(1)**:12–17, 2015.

47. Lešnik, S., Bertalan, E., Bren, U., and Bondar, A.-N.. Opioid receptors and protonation-coupled binding of opioid drugs. *International Journal of Molecular Sciences*, **22**:13353, 2021.
